# Combined fluorescence lifetime and surface topographical imaging of biological tissue

**DOI:** 10.1364/BOE.504309

**Published:** 2023-12-14

**Authors:** Charlotte Hopkinson, Andrew B. Matheson, Neil Finlayson, Michael G. Tanner, Ahsan R. Akram, Robert K. Henderson

**Affiliations:** 1 Institute for Integrated Micro and Nano Systems, School of Engineering, University of Edinburgh, Edinburgh EH9 3FF, UK; 2 Institute of Photonics and Quantum Sciences, School of Engineering and Physical Sciences, Heriot-Watt University, Edinburgh EH14 4AS, UK; 3 Centre for Inflammation Research, Institute of Regeneration and Repair, University of Edinburgh, Edinburgh BioQuarter, Edinburgh EH16 4UU, UK

## Abstract

In this work a combined fluorescence lifetime and surface topographical imaging system is demonstrated. Based around a 126 × 192 time resolved single photon avalanche diode (SPAD) array operating in time correlated single-photon counting (TCSPC) mode, both the fluorescence lifetime and time of flight (ToF) can be calculated on a pixel by pixel basis. Initial tests on fluorescent samples show it is able to provide 4 mm resolution in distance and 0.4 ns resolution in lifetime. This combined modality has potential biomedical applications such as surgical guidance, endoscopy, and diagnostic imaging. The system is demonstrated on both ovine and human pulmonary tissue samples, where it offers excellent fluorescence lifetime contrast whilst also giving a measure of the distance to the sample surface.

## 1. Introduction

Fluorescence imaging is a powerful technique for clinical applications as it can provide additional contrast between different tissue types compared to conventional white light imaging. Due to the auto-fluorescent behavior of many biomolecules when excited by UV or visible light, exogenous biomarkers are not always required. The simplest form of fluorescence imaging uses the intensity of emission to determine information about the fluorophores present. However, emission intensity can be greatly influenced by the excitation intensity, and fluorophore concentration. Widefield Fluorescence Lifetime Imaging (WFLIm) is a refinement of this technique and uses time resolved detection to make use of the characteristic lifetime of the fluorescence decay to provide additional contrast and a degree of independence from excitation intensity. WFLIm and scanning based fluorescence lifetime imaging have been shown to be valuable tools in surgical guidance [1], tumor identification [2–6] and tissue diagnosis, such as for identifying cardiovascular disease in arterial walls [7]. Additionally, work is ongoing to incorporate WFLIm into microendoscopy [8,9].

Another tool being used to enhance surgical guidance is 3D imaging, or surface mapping, which involves calculating the distance from the probe to the surface of the tissue. This has clear applications in endoscopy [10] and in surgical robotics [11], and the ability to better visualize the surface of tissue has been shown to improve instrument control during surgical procedures [10,12] and aid diagnosis of tissue abnormalities [13]. One way to generate the surface map is using time of flight (ToF) imaging. This method has been used extensively in applications such as autonomous vehicle control and mobile phone autofocus [14], but has also seen applications in endoscopic imaging [15]. Direct ToF imaging can be accomplished using fast, time resolved sensors to measure the round trip that a photon takes after scattering from an object, and thus its distance.

Both fluorescence lifetime, and ToF imaging require detectors with excellent time resolution and sensitivity. Time resolved single photon avalanche diodes (SPADs) meet both of these criteria. By operating the SPAD in time correlated single-photon counting (TCSPC) mode [16], in which a sensor measures the time of photon arrival relative to a pulsed laser source, both the time for a photon round trip and the fluorescent decay can be captured simultaneously. Using arrays of tens of thousands of SPADs on a single chip, widefield TCSPC imaging allows photons to be resolved spatially and avoids long imaging times associated with scanning based TCSPC technology [17]. SPADs have been used to perform widefield TCSPC imaging by providing high photon sensitivity and precise photon stamping capabilities [18], making them highly effective for both fluorescence lifetime [19–22] and distance measurements [23].

There is previous research showing techniques for combined ToF and fluorescence intensity [19,24] or fluorescence lifetime imaging [25–27]. These works all focus on subsurface imaging of phantoms or tissue with inclusions labelled with exogenous biomarkers that are excited in the deep-red or near infrared wavelength region. Hall et al. [24] describes a time resolved technique for calculating depth and fluorophore concentration of subsurface fluorescence in turbid medium using a scanning approach to gain spatial information. This was later improved upon by Han et al. [26,27] when a similar method was used to perform subsurface depth and lifetime imaging of mouse organs *in vivo*. Smith et al. [25] also demonstrates a method for calculating depth and fluorescence lifetime, however, liquid phantoms with fluorescent inclusions were used and results were improved using machine learning. Bruza et al. [19] were able to use the timing capabilities of a 2D SPAD array (SwissSPAD2) in order to calculate the depth of fluorophore labelled tumor tissue, although this time resolution was not used to provide fluorescence lifetime contrast.

Most recently, the authors of the current paper used a 32×32pixel SPAD array in TCSPC mode to perform combined fluorescence lifetime and ToF imaging [28]. This work differed from the previous techniques detailed above, as it aimed to determine the distance of the sample surface to the camera as opposed to subsurface depth, allowing surface mapping of the sample. The proof of principle work was conducted using a sensor with only 1024 pixels and much lower sensitivity than modern SPAD arrays, limiting its use for real life applications, but was still able to achieve < 1 cm distance and < 0.5 ns lifetime resolution over a 10 cm distance range. In this paper, the technique will be demonstrated using a more recent SPAD sensor with a 126×192pixel array and 40.6 ps time bin resolution [29,30]. To do this, visible light in the blue wavelength range is used as it penetrates only the very top surface of tissue (approximately 1 mm) [31]. A distance resolution of 4 mm is achieved while imaging 3D printed fluorescent targets which is an improvement compared to the previous work, as well as achieving a lifetime resolution of 0.4 ns. Additionally, images of ovine and human pulmonary tissue will be presented to show how the combined fluorescence lifetime and distance technique can be used on more biomedically relevant samples.

## 2. Experimental methods and materials

### 2.1. Optical setup

**Fig. 1.**
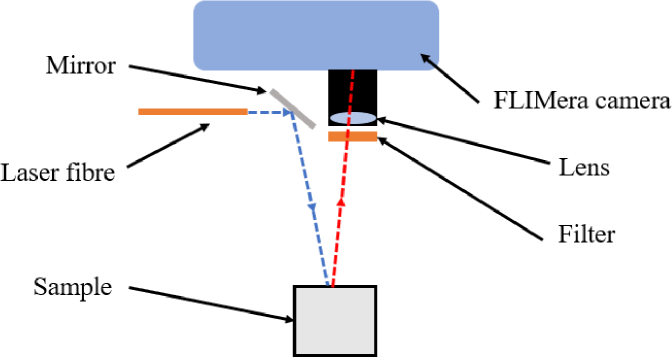
Schematic of the experimental setup.

A diagram of the setup is shown in Fig. 1. Fluorescence excitation is generated by a Hamamatsu Picosecond Light Pulser PLP-10 (wavelength of 483 nm, pulse width of 80 ps, maximum peak power of 150 mW and repetition rate of 50 MHz) which is coupled into an optical fiber (NA = 0.5, Thorlabs M124L02) and reflected onto the sample using a mirror. A 550 nm longpass filter (Thorlabs, FGL550M) is used to filter out scattered light and allow fluorescent photons to be collected by a machine vision lens (EFL = 12 mm, Navitar lens from Thorlabs, MVL12M23) which is focused onto the sensor giving approximately $16^{\circ }$ field of view. The experiments were performed in the absence of background light.

For these experiments, the TCSPC-based FLIMera camera by Horiba has been used, which is based around a 126×192 SPAD sensor array with 40.6 ps time bin resolution [29]. Photon histograms are generated in FLIMera’s fluorescence lifetime software (EzTime Image) where the histograms are then exported to allow for further analysis in MATLAB. SPAD exposure time was pre-set to approximately 80.9 s (1 million frames) for all images. Total acquisition time varied between 1 - 16 mins due to the data transfer rate, as the more photons detected by the sensor the longer the data transfer time. This time includes data processing in which photons are sorted into histogram bins and can be reduced by only saving the raw photon stamps.

### 2.2. Samples

Fluorescent filament targets were 3D printed into stepped structures using a high resolution 3D printer (Leapfrog Bolt Pro) to minimize any printing defects. The step size of the target is 0.5 cm, which was confirmed by measuring with vernier calipers, giving an error of less than 0.05 mm. Three commercially available filaments were used, each containing different fluorescent fluorophores to give distinct colors. One polylactic acid/polyhydroxyalkanoate (PLA/PHA) filament was used (colorFabb fluorescent green) and two PLA-only filaments were used (Real Filament fluorescent pink and Real Filament fluorescent orange). The supplier would not disclose the precise fluorophores they contain, but the fluorescence lifetimes calculated from the proprietary Horiba EzTime Image software were used as a baseline. The FLIMera camera and associated software are both commercially available and have previously been demonstrated to give highly accurate fluorescent lifetimes [29,32]. These filaments have previously been used in WFLIm using the same excitation and emission settings as are used in this work [9,28].

Ovine lungs were from ewes destined for cull and were euthanized under Schedule 1 of Animals (Scientific Procedures) Act 1986. Samples were frozen until required for experimentation.

Tumor and non-cancerous human lung tissue samples were obtained following approval by NHS Lothian REC and facilitated by NHS Lothian SAHSC Bioresource (REC No: 15/ES/0094), with informed consent from all patients. The tissue was assessed by a pathologist and areas of tumor were excised along with an additional sample of non-cancerous lung from the most distal part of the specimen resection. Pathology analysis demonstrated a poorly differentiated squamous cell carcinoma for the cancerous specimen and the non-cancerous lung demonstrating emphysematous changes.

## 3. Combined fluorescence lifetime and distance method

**Fig. 2.**
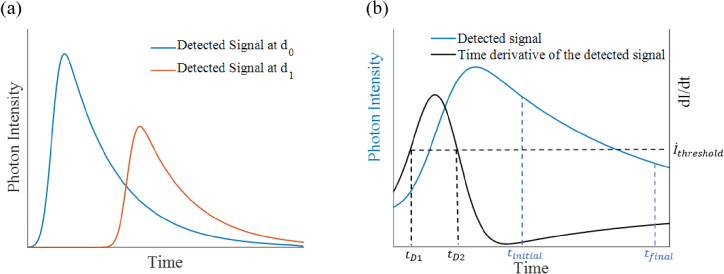
(a) Schematic of the detected signal at two different target distances. $d_{0}$ and $d_{1}$ refer to the target distance where $d_{0}<d_{1}$ . (b) Schematic of the detected signal and the time derivative of the detected signal. Position of the timings and intensity threshold applied in the combined fluorescence lifetime and distance method are represented by dotted lines.

When a target is moved further away from the sensor, the total distance a photon travels before it is detected increases. Eq. (1) shows the relationship between distance Δd and time of flight $t_{\text{ToF}}$ , which results in the time shift, and also a reduced intensity in the detected signal shown in Fig. 2(a). In Eq. (1), c is the speed of light. The rate of photon decay of the fluorescent target in Fig. 2(a) is described by the fluorescence lifetime τ and can be calculated using Eq. (2) where $I_{t}$ is the intensity at time t and $I_{0}$ is the peak intensity at $t=0\;(t_{0})$ .

(1) $$\Delta d=\frac{ct_{\text{ToF}}}{2}$$

(2) $$I_{t}=I_{0}\text{exp}\;\left(\frac{-t}{\tau }\right)$$

In these experiments, the scatter component of the detected signal is removed using a longpass filter to prevent SPAD pileup and the fluorescence getting overwhelmed, leaving only the fluorescence decay convolved with the instrument response function (IRF) of the signal [19]. The IRF of the FLIMera camera when using the Hamamatsu laser is found to be 0.44ns±0.13ns when the mean of the full width half maximum (FWHM) for each pixel is taken. SPAD arrays are susceptible to high dark count rates [29], where ’hot pixels’ can be seen as bright spots in the image. Background subtraction was performed on all data by taking an image in the absence of any light to remove these. To remove periodic noise in the data, the Matlab smoothdata function was used to perform an 8 point rolling average to the photon decays for each individual pixel. The remainder of this section presents a method to calculate both the fluorescence lifetime and the distance to the sample surface using the detected signal.

### 3.1. Fluorescence lifetime

The first step in calculating the fluorescence lifetime is to perform a least squares fit on the fluorescence decay. To do so we use a linearized form of Eq. (2), given in Eq. (3).

(3) $$\tau =\frac{-(t-t_{0})}{\text{ln}\;\left(\frac{I_{t}}{I_{0}}\right)}$$

The window we fit over is defined as being from $t_{\text{initial}}$ to $t_{\text{final}}$ which is shown schematically in Fig. 2(b). The time window which is fitted over can influence the calculated lifetime; fitting to very long time data may result in fitting to a noise floor resulting in an over-estimate of lifetime, fitting to early time data may result in fitting to residual scattered light or IRF limited decay resulting in an under-estimate of lifetime. It should be noted that for complex biological tissues the decay is unlikely to be mono-exponential and the precise window the fit is performed over will change the relative importance of each decay component, and thus the calculated value of τ . However, the aim of the WFLIm component of this work was simply to provide contrast between materials and tissue types, and so increasing the calculation speed at the expense of accuracy by using the linearized fit to Eq. (3) was deemed an acceptable compromise. Fluorescence lifetime values were calculated for each pixel in the array. The position of the fitting window (i.e., $t_{\text{initial}}$ to $t_{\text{final}}$ ) on the decay was determined by the time position of the peak fluorescence intensity. For filament samples with relatively long lifetimes, fits were performed where $t_{\text{initial}}$ and $t_{\text{final}}$ were 1 ns and 7 ns after the peak intensity respectively, resulting in a window with 148 time bins. For the tissue samples, much shorter lifetimes necessitated setting $t_{\text{initial}}$ and $t_{\text{final}}$ as 0.6 ns and 4.5 ns after the peak intensity respectively, resulting in a window with 95 time bins. This avoided the noise floor, at the expense of starting the time window closer to the IRF. To analyse the goodness of fit between the time points $t_{\text{initial}}$ and $t_{\text{final}}$ , $R^{2}$ values have been calculated for each pixel in the array, and these can be found in supplementary Fig. S1.

### 3.2. Distance using time of flight

Typically for ToF, Eq. (1) can be used to find the distance to an object’s surface. However, when the object is fluorescent there is the added complexity of the returning fluorescent signal being comprised of at least one exponential decay, such that it is no longer the same shape as the outgoing signal. This may result in an overestimation of the distance to objects with longer fluorescence lifetimes versus those with shorter lifetimes. To get around this problem, the ToF information can be gained from the rising edge of the returning signal, which is outlined as follows. The first step is to take the derivative of the fluorescence intensity with respect to time, to obtain $dI_{t}/dt$ . A threshold is then applied to the $dI_{t}/dt$ signal (shown as ${\dot{I}}_{\text{Threshold}}$ in Fig. 2(b)) and all data below the threshold is eliminated. In these experiments, the threshold was set to 50% which was found to give the most consistent distance results as evidenced by the plot of the standard deviation in distance for varying threshold values found in supplementary Fig. S2. As is shown schematically in Fig. 2(b), the resulting signal should have a narrow peak around the point of most rapid rise, which should be broadly independent of lifetime. A center of mass (CoM) calculation described in Eq. (4) is then used where $t_{\text{ToF}}$ is the time of flight for an individual pixel, the sums run between $t_{D1}$ and $t_{D2}$ , and $dI_{t}/dt$ is the first time derivative of the intensity at time t . The ToF can then be substituted back into Eq. (1) to obtain a distance for each individual pixel in the sensor array. We note that there is a time skew across the sensor, related to how long it takes for the clock signal to reach each pixel. This is corrected for in post-processing.

(4) $$t_{\text{ToF}}=\frac{\sum_{t=t_{D1}}^{t_{D2}}(dI_{t}/dt\cdot t)}{\sum_{t=t_{D1}}^{t_{D2}}(dI_{t}/dt)}$$

## 4. Results

**Fig. 3.**
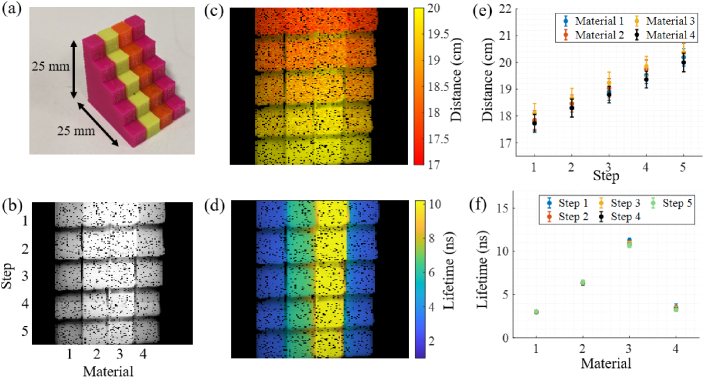
(a) Photograph of a fluorescent target made from different materials. (b) Intensity image of the front face of the target. The material changes in the x direction, and the step (distance) changes in the y direction. (c) Distance image of the scene in (b). (d) WFLIm image of the scene in (b). (e) Graph of the mean distance variation. (f) Graph of the mean lifetime variation between the materials.

Figure 3(a) shows a target made of four fluorescent objects that have been 3D printed into a stepped structure. This provides variation both in distance and lifetime. Two objects have been printed with fluorescent pink filament, one in fluorescent green, and one in fluorescent orange (the precise materials are described in Section 2.2). The individual objects have then been joined together to make the target. This target was imaged in TCSPC mode and the intensity, distance and fluorescence lifetime images are shown in Fig. 3(b), 3(c) and 3(d) respectively. An average of the photon decay for each material and step can be found in supplementary Figs. S3–S6.

The step size of the target is 0.5 cm, and this distance gradient is seen in Fig. 3(c), where the distance reduces for every row (in the y direction). An alpha map is applied to this image and all subsequent images, based on the intensity. In Fig. 3(c), 3(d) and all other subsequent images, distances and lifetimes in excess of 100 cm and 20 ns respectively are excluded as outliers. To assess how accurate the distance measurement is for each row and column, the mean distance and standard deviation has been calculated for each of the steps and this result is shown in Fig. 3(e). Some cross talk between material lifetime and distance estimation is observed, for example, the distance of material 3 (the material with longest lifetime) is slightly longer compared to the other materials. Longer lifetimes often lead to slower fluorescence rise time resulting in an over estimation of the distance. However, in these results the effect is small compared to the statistical variation of the distance across the fluorescent targets. In future, this could be addressed by taking the gradient of the rising edge into account. The maximum standard deviation, and therefore distance resolution of the sensor using the combined method is calculated to be 4 mm, similar to other time of flight based fibers [15]. It should be noted that this resolution will be partly dependant on the number of photon counts in the peak of the decay and the level of photon noise.

Excellent lifetime variation can be seen between the different materials as shown in Fig. 3(d). The outer materials were printed using the same fluorescent filament, and therefore show the same lifetime values, whereas the two central targets have differing lifetimes. For the fluorescence lifetime fit, $R^{2}$ values were calculated for every pixel and the mean was found to be 99.6% for all the pixels in Fig. 3(d), as shown in supplementary Fig. S1. Mean lifetimes and standard deviation for each row and column have been calculated and shown in Fig. 3(f). The fluorescence lifetimes calculated using the combined fluorescence lifetime and distance method are in excellent agreement with those which have been calculated using the EzTime Image software and found to be approximately 6.1 ns, 2.7 ns and 10.1 ns for the green, pink and orange filaments respectively. By comparing Fig. 3(b) and 3(d), we can see that fluorescence lifetime provides enhanced contrast compared to intensity alone. The maximum standard deviation, and therefore lifetime resolution of the sensor using the combined method is calculated to be 0.4 ns.

Having demonstrated the effectiveness of the system on highly fluorescent test targets, more complex and biomedically relevant materials could be imaged.

**Fig. 4.**
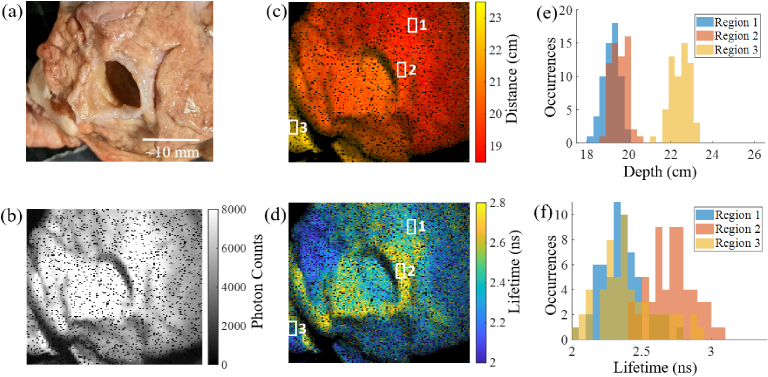
(a) Photograph of sheep lung. (b) Intensity image of the scene in (a). (c) Distance image of the scene in (a). (d) WFLIm image of the scene in (a). (e) Histogram of the distance variation in three regions outlined in (c). (f) Histogram of the lifetime variation in the three regions outlined in (d).

Figure 4(a), 4(b), 4(c) and 4(d) show a photograph, photon intensity, distance and lifetime images of a section of sheep lung. Individual photon decays for a range of pixels for the sheep lung images can be found in supplementary Fig. S7(a)-(c), including details of the lifetime fit. The surface of the sheep lung tissue is at an angle where the top right side of the sample is closer to the camera than the bottom left side, and this is clearly shown in the distance map in Fig. 4(c). Three regions have been selected to compare distance and lifetime across the sample and are identical in position and size in both Fig. 4(c) and 4(d). In Fig. 4(e) and 4(f) histograms have been generated to show the three regions. Figure 4(e) shows a shift in the calculated distance as the tissue gets further away from the camera since Region 3 is shown to be further from the camera than Regions 1 and 2. The fluorescence lifetime image and histogram in Fig. 4(d) and 4(f) show areas of longer lifetime in the region around the hole in the tissue (remnant of a major airway). Additionally, some areas on the bottom left of Fig. 4(d) are shown to have longer lifetime. These areas have been identified as fatty tissue (compared to the rest of the sample) which has been shown in literature to have a longer lifetime compared to other tissue types [33]. For the fluorescence lifetime fit, $R^{2}$ values were calculated for every pixel and the mean was found to be 93.5% for the sheep lung tissue.

**Fig. 5.**
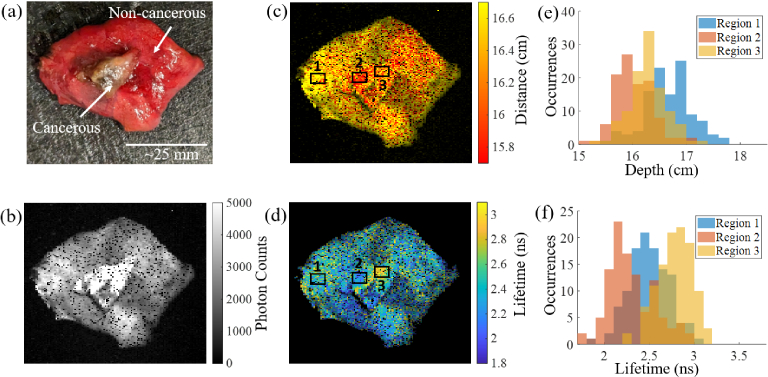
(a) Photograph of human lung cancer tissue positioned on top of a non cancerous piece of lung tissue. (b) Intensity image of the scene in (a). (c) Distance image of the scene in (a). (d) WFLIm image of the scene in (a). (e) Histogram of the distance variation in three regions outlined in (c). (f) Histogram of the lifetime variation in the three regions outlined in (d).

To show that the combined distance and lifetime method is applicable to detecting abnormalities in human tissue, lung tissue samples removed from patients undergoing surgical resection for lung cancer have been imaged. Both non-cancerous and cancerous tissue have been analysed and photographs, intensity, distance and lifetime images of these are shown in Fig. 5(a), 5(b), 5(c) and 5(d) respectively. Individual photon decays for a range of pixels for the human lung images can be found in supplementary Fig. S8(a)-(c), including details of the lifetime fit. The tissue samples were placed with the cancerous tissue on top of the non-cancerous tissue in a petri dish in order to give some distance variation. Three regions have been selected to compare different distances and lifetimes, and are identical in position and size for both Fig. 5(c) and 5(d). Fig. 5(c) and the histograms in Fig. 5(e) show the distance variation, where Regions 2 and 3 are shown to be closer to the camera compared to Region 1. Figures 5(d) and 5(f) indicate that areas of the smaller piece of tissue may have a longer lifetime to the larger piece. This gives the first indication that a combined ToF and lifetime based system may be viable in applications involving human cancerous tissue. For the fluorescence lifetime fit, $R^{2}$ values were calculated for every pixel and the mean was found to be 84.6% for the human lung tissue. It should also be noted, that in Fig. 5(b) and 5(d) the materials are exhibiting endogenous fluorescence, without the need for any additional dyes or biomarkers. No attempt has been made to try and identify the precise endogenous fluorophores within the human lung tissue which are being excited. Fluorescent molecules associated with cancerous and non-cancerous tissue are complex, and can vary greatly depending on location within the tissue, and the tissue itself, however, there are other works that aim to explore the properties of cancerous tissue using fluorescence [34]. Both Fig. 5(c) and 5(d) have high levels of noisy pixels compared to the ovine lung and fluorescent filament samples, the reason for this being that the sample is overall less fluorescent, giving larger distance and lifetime variability across the sample. In future this could be improved using higher laser powers, more efficient emission and collection optics, and the addition of fluorescent dyes.

## 5. Conclusion

The results presented here represent the first demonstration of combined WFLIm and surface topographical imaging of biological tissue, let alone human tissue with a known pathology. They demonstrate significant potential in diagnostic imaging, surgical guidance and microendoscopy, although the 1-16 min acquisition times are not yet suitable for *in vivo* imaging. However, there is clear scope for improving on this in future work. The 150 mW excitation power is relatively modest, and could be increased using a more powerful laser or more efficient optical fiber. Additionally, the laser wavelength used in this work is not necessarily optimal for exciting endogenous fluorophores. By using shorter wavelength excitation (for example 400 nm) we would expect significantly more emission from fluorophores such as flavins [34]. Future work will also benefit from using a new generation of SPADs with improved photon detection efficiency, improved fill factor [35] and lower dark count rates [36]. By combining all these improvements, it should be possible to significantly improve the photon budget and thus reduce the necessary exposure time. Finally, our group has previously achieved high speed fluorescence lifetime imaging using camera systems where the lifetime calculation is carried out on Field Programmable Gate Arrays (FPGA) rather than in software [37]. Having demonstrated the effectiveness of our combined distance and lifetime technique, it may be possible to shift much of the calculations from software to FPGA and thus significantly increase the data processing step. Carrying out the above improvements to speed up both the data acquisition and processing, should allow us to move closer to real-time imaging.

## Data Availability

Data underlying the results presented in this paper are available in Ref. [38]

## Supplemental document

See Supplement 1 for supporting content.
