# Supplementary material for: Combined fluorescence lifetime and surface topographical imaging of biological tissue
Source: Biomed Opt Express. 2023 Dec 14;15(1):212–21. doi: 10.1364/BOE.504309 (PMC10783922; doi:10.1364/BOE.504309)
Supplement: Supplementary file 1 [file boe-15-1-212-s001.pdf]

Supplemental Document

Biomedical Optics EXPRESS

Combined fluorescence lifetime and surface topographical imaging of biological tissue: supplement

CHARLOTTE HOPKINSON,[1] ANDREW B. MATHESON,[1] NEIL FINLAYSON,[1] MICHAEL G. TANNER,[2] AHSAN R. AKRAM,[3] AND ROBERT K. HENDERSON[1,*]

[1] *Institute for Integrated Micro and Nano Systems, School of Engineering, University of Edinburgh, Edinburgh EH9 3FF, UK*
[2] *Institute of Photonics and Quantum Sciences, School of Engineering and Physical Sciences, Heriot-Watt University, Edinburgh EH14 4AS, UK*
[3] *Centre for Inflammation Research, Institute of Regeneration and Repair, University of Edinburgh, Edinburgh BioQuarter, Edinburgh EH16 4UU, UK*
[*] *robert.henderson@ed.ac.uk*

This supplement published with Optica Publishing Group on 14 December 2023 by The Authors under the terms of the Creative Commons Attribution 4.0 License in the format provided by the authors and unedited. Further distribution of this work must maintain attribution to the author(s) and the published article's title, journal citation, and DOI.

Supplement DOI: https://doi.org/10.6084/m9.figshare.24512599

Parent Article DOI: https://doi.org/10.1364/BOE.504309

Combined fluorescence lifetime and surface topographical imaging of biological tissue: supplemental document

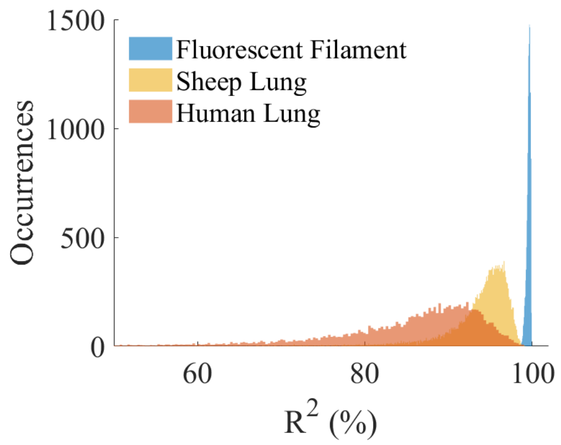

Fig. S1. Histograms of R^2 values to show the goodness of fit of the curve used to estimate fluorescence lifetime. This has been done for the three datasets (fluorescent filament, sheep lung tissue and human lung tissue).

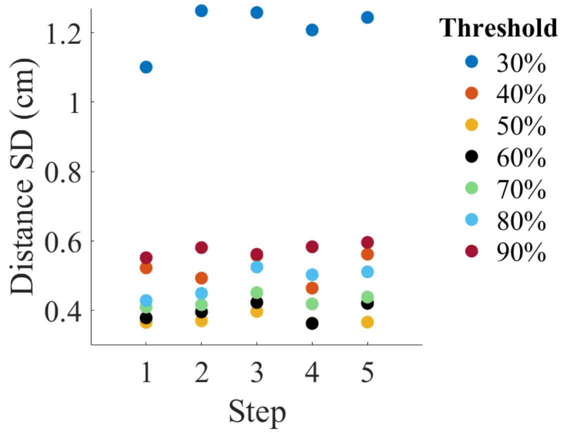

Fig. S2. Plot of standard deviation (SD) in distance across the pixels for Step 1-5 of Material 3, calculated using different threshold values.

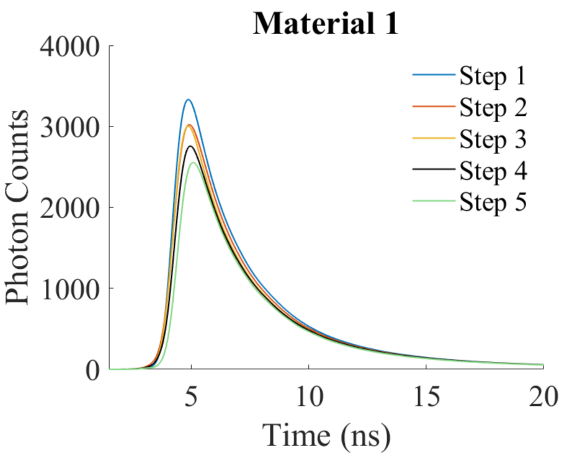

Fig. S3. Photon decay curves calculated by taking an average over the pixels for each step of Material 1.

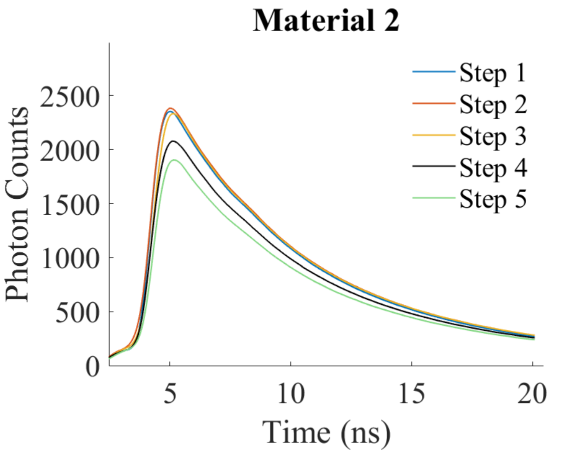

Fig. S4. Photon decay curves calculated by taking an average over the pixels for each step of Material 2.

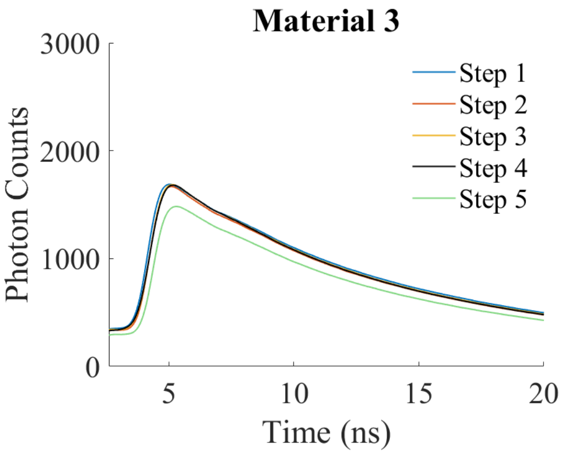

Fig. S5. Photon decay curves calculated by taking an average over the pixels for each step of Material 3.

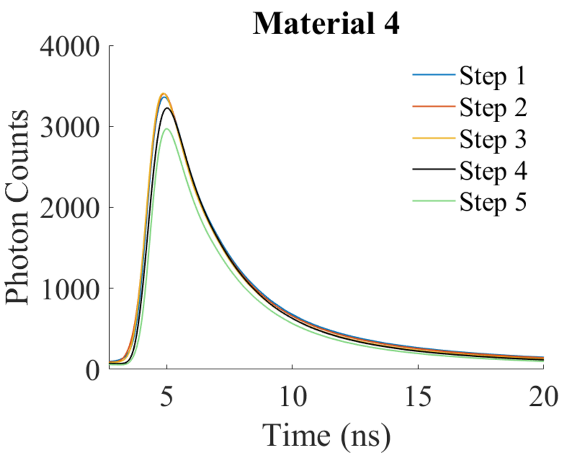

Fig. S6. Photon decay curves calculated by taking an average over the pixels for each step of Material 4.

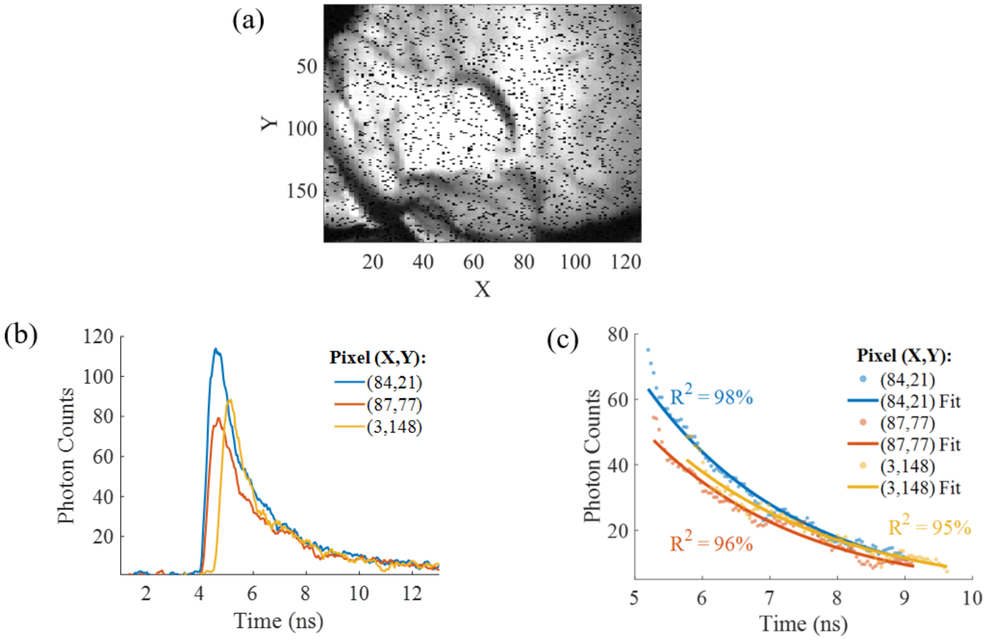

Fig. S7. (a) Intensity image of the sheep lung tissue with pixel number shown on the X,Y axis. (b) Photon decays of three pixels from (a). (c) As (b), but showing only the range between $t_{initial}$ and t_{final}, along with the fits to Eq(3). R^2 values of fits are shown alongside.

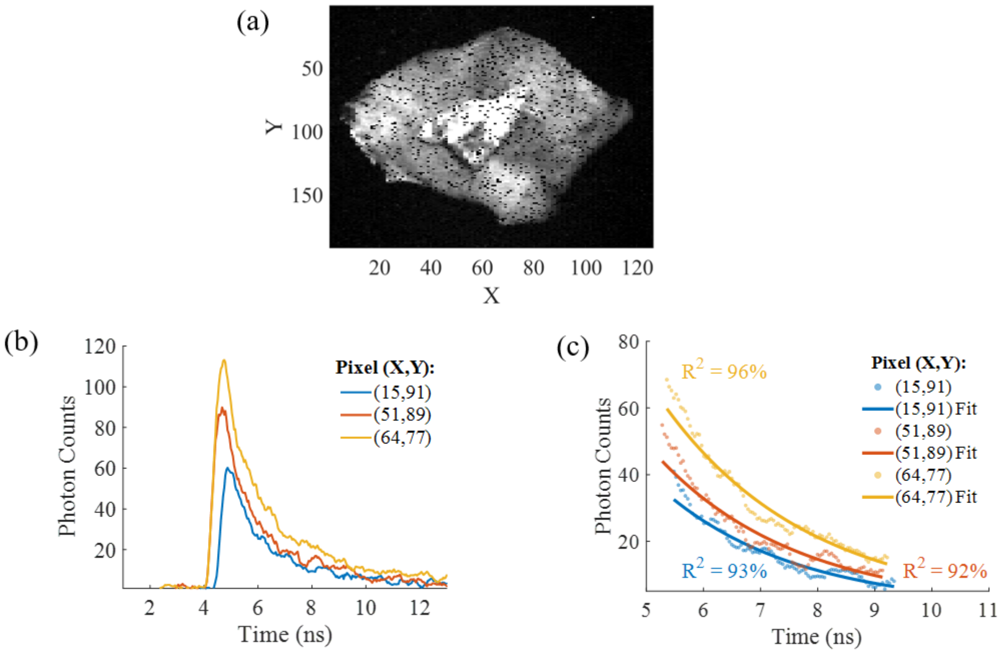

Fig. S8. (a) Intensity image of the human lung cancer tissue positioned on top of a non cancerous piece of lung tissue with pixel number shown on the X,Y axis. (b) Photon decays of three pixels from (a). (c) As (b), but showing only the range between $t_{initial}$ and t_{final}, along with the fits to Eq(3). R^2 values of fits are shown alongside.
